# A Trimodality, Four-Step Treatment including Chemotherapy, Pleurectomy/Decortication and Radiotherapy in Early-Stage Malignant Pleural Mesothelioma: A Single-Institution Retrospective Case Series Study

**DOI:** 10.3390/cancers14010142

**Published:** 2021-12-29

**Authors:** Giovanni Vicidomini, Carminia Maria Della Corte, Antonio Noro, Raimondo Di Liello, Salvatore Cappabianca, Alfonso Fiorelli, Valerio Nardone, Gaetana Messina, Giuseppe Viscardi, Angelo Sangiovanni, Riccardo Monti, Marina Accardo, Floriana Morgillo, Fortunato Ciardiello, Renato Franco, Mario Santini

**Affiliations:** 1 Thoracic Surgery Unit, Department of Translational Medicine, University of Campania “Luigi Vanvitelli”, 80131 Naples, Italy; antonio.noro@unicampania.it (A.N.); alfonso.fiorelli@unicampania.it (A.F.); gaetana.messina@unicampania.it (G.M.); mario.santini@unicampania.it (M.S.); 2 Medical Oncology Unit, Department of Precision Medicine, University of Campania “Luigi Vanvitelli”, 80131 Naples, Italy; carminiamaria.dellacorte@unicampania.it (C.M.D.C.); raimondo.diliello@unicampania.it (R.D.L.); giuseppe.viscardi@unicampania.it (G.V.); floriana.morgillo@unicampania.it (F.M.); fortunato.ciardiello@unicampania.it (F.C.); 3 Division of Radiodiagnostic, Department of Precision Medicine, University of Campania “Luigi Vanvitelli”, 80138 Naples, Italy; salvatore.cappabianca@unicampania.it (S.C.); valerio.nardone@unicampania.it (V.N.); angelo.sangiovanni@unicampania.it (A.S.); riccardo.monti@unicampania.it (R.M.); 4 Pathology Unit, Department of Mental and Physical Health and Preventive Medicine, University of Campania “Luigi Vanvitelli”, 80138 Naples, Italy; marina.accardo@unicampania.it (M.A.); renato.franco@unicampania.it (R.F.)

**Keywords:** mesothelioma, surgery techniques, chemotherapy, radiation therapy, statistics, survival analysis

## Abstract

**Simple Summary:**

Multimodality treatment can improve outcome of malignant pleural mesothelioma (MPM) patients. However, the ideal scheme of combination of them is still unknown. We analyzed a case series of 17 patients treated at our institution with a sequence of induction chemotherapy, surgery, adjuvant radiotherapy and chemotherapy. Median overall survival was 32.1 months, median progression free survival was 23.7 months with a safe profile. These data according to our experience represent a great example of feasibility in clinical practice of three-modality four step approach and encourage further prospective studies to better define the details of treatment.

**Abstract:**

Background: Multimodality treatment is considered the best treatment strategy for malignant pleural mesothelioma (MPM). However, the ideal combination of them is still a matter of controversy. Here, we report a case series of MPM treated with a trimodality approach: induction chemotherapy (CT), pleurectomy/decortication (P/D), postoperative radiotherapy (RT) and post-operative CT. Methods: A retrospective case series of 17 MPM patients treated between 2013 and 2020 is presented. Patients had epithelial or mixed MPM diagnosed by video-assisted thoracoscopy and pathologic IMIG stage I or II disease. Treatment details and radiological data were collected. Induction therapy consisted of combination of cisplatin and pemetrexed, every 21 days for two cycles. P/D was performed 4–6 weeks after induction CT, post-operative RT 3–6 weeks after surgery, while post-operative CT was given 4–6 weeks after RT, with the same schedule of induction. Results: All patients showed objective response or stability of disease at the restaging following induction CT and underwent surgery by posterolateral thoracotomy. There were two cases of cardiac arrest as major intraoperative complication, both resolved by manual cardiac massage. Minor complications included one hemidiaphragm elevation, 1 anemia requiring blood transfusion, one wound infection, and two persistent air leaks. Median overall survival was 32.1 months, median progression free survival was 23.7 months. Conclusions: These results suggest the feasibility of these trimodality treatment scheme for early stage MPM patients. Larger series and long-term prospective studies are needed to confirm the validity of this strategy.

## 1. Introduction

Malignant pleural mesothelioma (MPM) is an invasive, locally aggressive tumor that is related to professional asbestos exposure [1]. Unfortunately, it is almost always fatal, and its incidence is expected to increase at least for the next decade. Conservative therapy of MPM results in median survival of about 12 months after diagnosis [2]. Its aggressiveness and treatment resistance lead to a typical tumor progression in the chest that is manifested by increasing mass size and pleural effusion, with unavoidable compression of the ipsilateral lung and consequent respiratory function deterioration [3]. Although distant metastasis can occur with advanced disease, the inadequate local control usually is the main cause of death.

Despite numerous therapeutic attempts, there is still no cure or any evidence of the optimal treatment. In the past, several single-modality therapies have been applied, showing disappointing results [4]. For this reason, researchers have begun to evaluate different multimodal therapeutic approaches, especially in early-stage disease, combining the various possible treatments. Multimodality therapy seems to be the best treatment for malignant pleural mesothelioma but the ideal combination of currently adopted therapies is still undefined and few prospective randomized trials are reported in literature [5,6,7].

Even the role of surgical resection in the management of MPM is still a matter of controversy [8,9]. The most used surgical techniques are extrapleural pneumonectomy (EPP), pleurectomy/decortication (P/D), and extended P/D (EP/D) [10,11,12]. There are no established guidelines; thus, the choice among these three procedures depends not only on the patient performance status (PS), tumor stage and intraoperative findings but also on the surgeons’ experience. EP/D competes against EPP as surgical therapy modality with the aim to achieve macroscopic complete resection. Originally P/D was a palliative option for controlling pleural effusion, but currently it seems to be a valid alternative to EPP in a multimodality therapy model [13,14].

We present a case series of 17 stage I and II MPM patients treated at our institution with a trimodality, four-step approach: induction chemotherapy (CT), P/D, postoperative radiotherapy (RT) and postoperative CT.

## 2. Patients and Methods

### 2.1. Patients’ Selection and Data Collection

The case series was collected retrospectively. Patients’ data were collected consecutively over time from internal database, among 97 patients who received the diagnosis of MPM at our institution from 2013 to 2020. We identified 17 patients who completed a “trimodality therapy”, including two cycles of induction CT followed by P/D, post-operative RT, as well as post-operative CT (see CONSORT diagram, Figure S1) and we collected clinical data.

Any decision regarding therapy had been first discussed in a multidisciplinary thoracic oncology team and then with the patients.

All patients had a histological diagnosis of epithelial or biphasic MPM obtained with a video-assisted thoracoscopy performed in our Institution and a pathologic International Mesothelioma Interest Group (IMIG) stage I or II disease.

For all patients, we collected clinical history and physical examination, laboratory data, imaging assessing, pulmonary function testing (spirometry, arterial blood gas analysis) and echocardiography. Toxicity data were retrospectively categorized according to Common Terminology Criteria for Adverse Events (CTCAE) v5.0. Data collection for this case series was approved by local Ethical Committee.

### 2.2. Objectives

The primary objective of this case series collection was to assess the feasibility and clinical outcome of radical surgery in MPM patients, with potentially resectable staging after two cycles of CT, followed by RT and further two cycles of CT. 

**Figure 1:**
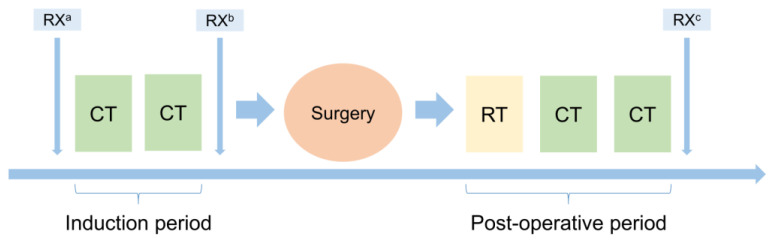
Protocol scheme. RX: baseline ^a^, post-induction ^b^ and first follow-up ^c^ radiological evaluation; CT: chemotherapy; RT: radiotherapy.

The therapeutic scheme proposed is shown in Figure 1. Induction therapy consisted of combination of cisplatin and pemetrexed, every 21 days for 2 cycles. P/D was performed 4–6 weeks after induction CT, post-operative RT 3–6 weeks after surgery, while post-operative CT was given 4–6 weeks after RT, with platinum-pemetrexed combination. Clinical outcome collected were overall survival (OS), progression free survival (PFS), pattern of response and recurrence.

### 2.3. Statistical Methods

Continuous variables were expressed in terms of median values and range, while categorical variables were presented as frequencies and percentages. PFS was defined as the time from diagnosis to the first event among death (any cause) or progression of disease. OS was defined as the time from diagnosis until death (any cause) and was estimated by the Kaplan–Meier method. PFS and OS between groups was compared with unadjusted log-rank test. Statistical analyses were performed with IBM^®^ SPSS/PASW software v23.0 (SPSS, Inc., Chicago, IL, USA).

## 3. Results

A total of 17 MPM patients were identified. The patients’ characteristics are showed in Table 1. Median age was 66 years (range 58–73), with prevalence of male gender (70.6%), baseline Eastern Cooperative Oncology Group (ECOG) PS of 1 (58.8%) and epithelial histology (88.2%). Median follow-up was 43.4 months.

### 3.1. Induction Therapy

Induction CT consisted of 2 cycles of combination of cisplatin (75 mg/m^2^ on day 1) and pemetrexed (500 mg/m^2^ on day 1) every 21 days in all patients, with good tolerability, without any grade 3–4 adverse events. Disease was re-assessed radiologically with total body CT scan, magnetic resonance imaging (MRI) of chest and fluorine-18 (^18^F) fluorodeoxyglucose (FDG) positron emission tomography (PET). No complete responses were observed, with evidence of metabolic response in 10/17 (58.8%) of patients.

### 3.2. Surgery

All patients underwent surgery by posterolateral thoracotomy. P/D included resection of parietal and mediastinal pleura and removal of the visceral pleura, without resection of the lung. Isolated lung ventilation was used. Posterolateral thoracotomy was performed in the sixth intercostal space. The extrapleural dissection from the chest wall and mediastinum was done *en-bloc* till the hilum was reached. Afterwards, the visceral pleura was dissected applying positive end-expiratory pressure on the lung operated on, if necessary. The visceral pleura of the fissures was also dissected. In case of evidence of resectable small lesions over diaphragm and pericardium, they were selectively removed; in case of grossly invasion of diaphragm or pericardium, they were extensively resected. The diaphragm defect was always reconstructed with a synthetic mesh (prolene polypropylene mesh or bovine pericardium mesh) placed in the anatomic position of the resected portion to minimize radiation exposure to abdominal contents. The pericardium defect was reconstructed in a similar manner. In particular, the surgical procedure was extended in five patients with partial resection and reconstruction of the diaphragm; three of these patients had concomitant partial resection and reconstruction of pericardium (stage II patients). In addition, in two cases pulmonary wedge resections were also performed for peripheral involvement of the parenchyma by mesothelioma. There were two major intraoperative complications: two patients had intraoperative cardiac arrest, resolved by manual cardiac massage. We observed one case of hemidiaphragm elevation after resection and subsequent prosthetic reconstruction, that did not require reoperation. Minor post-operative complication was evidenced in other four patients: anemia requiring blood transfusion in one patient, wound infection in one, and persistent air leaks in two. No post-operative mortality was observed. Twelve of seventeen patients (70.6%) obtained a complete microscopically margin-negative resection (R0/R1), whereas five (29.4%) showed residual disease at the following imaging examinations (R2). In those R2 patients, mean residual tumor mass at CT scan after surgery was 10% (range 3–20%).

### 3.3. Post-Operative RT

For planning purpose, a CT planning scan was performed with patients immobilized lying supine, arms overhead. To take organ and target motions into consideration, 4D-CT was used. The PTV (planning target volume) was contoured as a ring-shaped area that included the intercostal muscles, the pre-surgery pleural surface, the ribcage thickness, the chest wall as well as the surgical scars. Inter-lobar pleura was excluded, as well as the skin superficial surgical scars. The whole pleura was included adding a 3 mm margin to the inner surface of the PTV. The cranial margin was 1.5 cm above the apex of the lung, whereas the posterior-lateral and front-lateral borders overlapped the vertebral bodies and the sternum. On the medial side the PTV encompassed the ipsilateral pericardium. The lower limit included the whole diaphragm until bone insertion (close to L2 vertebral body).

The total dose was 50 Gy delivered in 25 fractions and the prescribed isodose to PTV was 95%. A boost of 10 Gy was delivered to cover residual disease (R2) patients (five patients, 29.4% of the cohort).

Mandatory dose-volume constraints for organs at risk (OARs) to be eligible for post-operative radiotherapy included volume of contralateral lung receiving 5 Gy (V5) < 17% and Maximal dose (D max) to the spinal cord < 50 Gy. Secondary OARs constraints included total lung dose < 24 Gy, ipsilateral and contralateral kidneys V25 < 40% and V10 < 10%, liver V30 < 40%. Post-operative RT started 3 to 6 weeks post-surgery. The patients underwent radiotherapy with volumetric modulated arc therapy (VMAT). The median dose of postoperative RT was 50 Gy (range, 45.1–50.4 Gy).

Postoperative external beam radiation therapy caused no ≥ G3 complication, whereas G2 pneumonitis was observed in 8 patients (47% of the whole cohort) and G1 pneumonitis in 13 patients (76%). All the symptomatology was rapidly resolved with conservative management.

### 3.4. Post-Operative CT

Post-operative CT was done 4–6 weeks after RT in all patients with cisplatin or carboplatin (according to clinical conditions) and pemetrexed, with the same schedule of induction. A total of 2 cycles of CT were administered in 100% of patients, again without any occurrence of ≥G3 toxicity.

### 3.5. Follow-Up and Efficacy Outcomes

**Figure 2:**
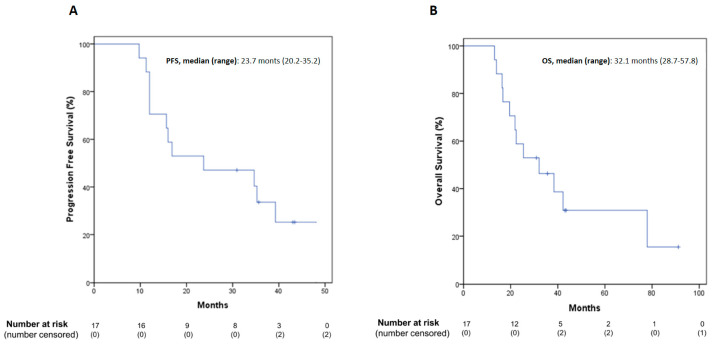
PFS (**A**) and OS (**B**) Kaplan–Meier curves in the total population. PFS: progression-free survival; OS: overall survival.

**Figure 3:**
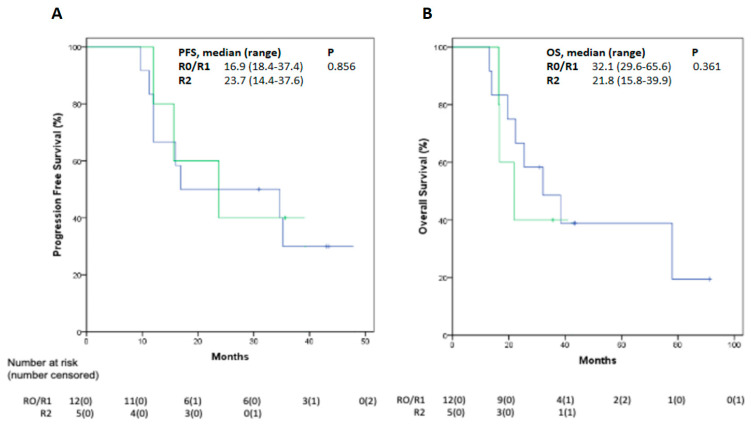
PFS (**A**) and OS (**B**) Kaplan–Meier curves by residual disease (R0 vs. R1/R2). PFS: progression-free survival; OS: overall survival.

Serial chest CT scan and abdomen ultrasound imaging were performed every 3 to 4 months after the end of post-operative treatment. All patients were followed up until death or to the final date of analysis for the present study. Recurrences occurred in 14/17 patients (82%). The site of recurrence was mostly loco-regional along the previous site of surgically resected pleural disease (12/14, 86%), while distant metastases were detected in two patients (one in the contralateral lung and one in the spinal bones). Median PFS was 23.7 months (range 20.2–35.2 months, Figure 2A). At the time of data cut-off, 5/17 patients were still alive (29.4%). Among patients experiencing disease recurrence, eight (47.1%) were treated with palliative systemic therapy. Four patients received platinum-based CT as rechallenge strategy, four patients were treated with single-agent CT. Median OS was 32.1 months (range 28.7–57.8 months, Figure 2B). All deaths were correlated to disease progression. No significant differences were observed comparing PFS between R0/R1 and R2 patients, but an interesting trend of OS increase was found in R0/R1 subgroup (32.1 months versus 21.8 months in R2 patients, Figure 3). Moreover, we compared PFS and OS in stage IA patients (*n* = 5) versus all other patients (*n* = 12): both PFS and OS are still not reached in MPM patients with early stage (IA) (Figure S2) versus 16 months and 25.4 months in the other patients. Additionally, in the 5/17 patients who underwent extended resection of hemidiaphragm or pericardium, median PFS and OS were 39.3 and 42.2 months, respectively.

## 4. Comment

MPM is a rare neoplasm associated with a previous exposure to asbestos; it has been considered for a long time invariably fatal and intractable. The main problem is to obtain a local control of the disease, since the aggressiveness of the neoplasm leads to rapid involvement not only of the entire pleura but also of the chest wall, diaphragm, and mediastinum. For this reason, it is often diagnosed in a locally advanced stage, when it is already not susceptible to curative radical surgical approach [2]. Furthermore, several recent studies showed that OS is much longer than previously thought if the malignancy is diagnosed in the early stages. Treatment options for MPM today include surgical resection, CT, RT, and recently also new medical treatments, like immunotherapy, according to stages and clinical conditions of any single patient [7].

Regarding surgery, it has not proved sufficient alone in eradicating all local disease and a high likelihood of local recurrence and R2 resection [15,16]. For this malignancy, the main goal in a multimodal strategy is to obtain a complete removal of all macroscopic tumor tissue (cytoreductive surgery), thus improving the effectiveness of subsequent treatments. The two surgical options currently most used are EPP and P/D. The aggressiveness of the EPP led to an increase in mortality and morbidity if compared with P/D. In the trial by Treasure et al., the authors compared the effects of EPP plus postoperative hemithoracic RT versus standard (non-radical) therapy alone following platinum-based CT in the MARS 1 study. The trial included 50 patients, results suggested that a radical approach with EPP offered no advantage over a non-radical approach [17]. This hypothesis is confirmed by Bölükbas et al. who support P/D as an option within the multimodal regimen with promising results in terms of long-term survival, morbidity, and mortality. Furthermore, full delivery of trimodality therapy seems more feasible with P/D than with EPP [18].

Therefore, the therapeutic approach for MPM has changed over the past 20 years and the prominence of aggressive EEP has largely been abandoned in favor of P/D, a more conservative surgical approach within a multimodal treatment [5]. Currently, several ongoing trials are evaluating the effects of multimodal therapies: MARS 2 is a randomized controlled trial that compares the outcomes of platinum-based CT plus P/D versus CT alone [19]. This trial differs from the previously discussed ones since it includes P/D instead of EPP as surgical treatment [19].

Anyway, a part from randomized trials, several retrospective and prospective cohort studies or cancer registry reports suggested an advantage for patients undergoing surgery [20,21]. Despite these data should be considered with caution, they showed that trimodality treatment could represent the best combination and realistic opportunity to prolong OS in this population [2]. In the recent past, the combination of neoadjuvant CT, P/D, and adjuvant pleural intensity modulated radiation therapy (IMRT) or VMAT has shown to be a promising multimodality treatment paradigm [22,23]. Regarding CT, cisplatin and pemetrexed has been adopted as the standard frontline regimen from decades. The EORTC 1205 trial, a phase II randomized trial comparing an immediate surgery arm consisting of P/D followed by three cycles of cisplatin/pemetrexed with delayed surgery starting with the same chemotherapeutic regimen followed by P/D, suggesting a potential role for surgery after induction therapy [24].

These data suggest that survival is more promising with multimodal treatment, but the optimal type, combination, and timing of the most widely used treatment options, such as surgery, RT, and CT, have yet to be established. Another issue to consider is the lack of predictive factors for patient selection outside clinical ones as the stage of the disease and the PS of patients. Preclinical evidences suggest that sensitivity to CT, especially platinum-based, could be related with BAP-1 status [25,26]. Notwithstanding, the use of this biomarker for treatment allocation is not recommended outside of clinical trials. BAP-1 mutations, that occur in 25–60% of the cases has been also evaluated as a possible biomarker of poly(ADP-ribose) polymerase (PARP) inhibitors efficacy in the single arm MiST-1 trial but conclusive results are awaited [27].

In this manuscript, we present a case series of 17 MPM patients treated with a multimodal approach. In clinical practice at our institution, MPM patients are always treated by multi-disciplinary group. All patients presented underwent macroscopically radical surgery after two cycles of CT, followed by RT and further two cycles of CT. Induction CT was well tolerated and effective in controlling disease. Regarding RT, its use after lung-sparing techniques such as P/D has always been questioned due to risk of severe lung toxicity. The discouraging results of several studies based on hemithoracic radiation prompted the investigation of novel radiation delivery techniques to improve local control [28]. RT schemes involving irradiation of the thoracic wall sparing the underlying healthy lung, may represent a safe option [29].

In our case series, no severe toxicity was observed in patients treated with VMAT, with no patients showing ≥G3 toxicity. It is noteworthy to underline, at this regard, that the total dose was limited to 50Gy in the majority of our patients, and the volume of the boost was limited to include only residual disease, with no patients showing a big burden of residual disease requiring higher doses.

At the same time, in our case series, no treatment related mortality occurred. Specifically, we did not observe any postoperative deaths at either 30 or 90 days, as opposed to other studies using EPP as surgical approach [30]. Surgical complications occurred in 7/17 patients (41.2%) with only 2 major ones without fatal evolution. The major complications included two cardiac arrests resolved by manual cardiac massage. Interestingly, this complication occurred in two patients undergoing to pericardial resection. Induction and postoperative CT and RT also showed no severe toxicity. The median OS of the entire cohort was 32.1 months and the median PFS was 23.7 months, that can be positively compared to different experiences reported in the literature [31]. As an example, Bölükbas et al. found a median survival of 30 months, evaluating 35 patients undergoing P/D followed by adjuvant CT and RT [18]. However, we believe that addition of induction CT could reduce disease burden and select chemo-responsive patients for surgery. In this regard, our data are encouraging, as compared to literature data for similar patients not re-assessed and not undergoing surgery after two cycles of induction of CT, suggesting a potential efficacy of this treatment approach [32]. Furthermore, our comparison of PFS and OS in stage IA patients versus all other patients suggest that multi-modality treatment could be particularly effective in early stages.

Most likely, the idea of limiting the surgical procedure, thus preserving the underlying lung, was a key component of the promising survival observed. Other studies with good survival outcomes using EPP showed significantly higher mortality and complications [6,33]. In this regard, data on our patients undergoing extended P/D (i.e., pleurectomy/decortication associated with resection of the pericardium and/or diaphragm) suggest that this intervention may improve the clinical outcome in patients with macroscopic invasion of diaphragm and pericardium. Finally, in our patients the proposed therapeutic protocol demonstrated to preserve respiratory function and hence quality of life. Specifically, we speculate that the administration of the minimum number of chemotherapy cycles (at least two) may positively impact the clinical conditions of patients for surgery. The radiological evaluation after two cycles guaranteed that macroscopic radical surgery was feasible, followed by positive local anti-tumor effect of RT. Moreover, the additional two adjuvant cycles of chemotherapy could be particularly useful to prevent recurrence. Recently published MPM guidelines [2] are still inconclusive for standardization of multimodal treatments, still recommending peri-operative platinum-pemetrexed chemotherapy, with timing to be defined (NCT02436733 trial).

Although many authors have questioned the value and role of surgery in the treatment of MPM, we believe that further studies should be conducted to confirm the potential efficacy and tolerability of this four-step treatment approach.

Certainly, the presentation of a case series of patients is affected by many limitations. Firstly, the retrospective and descriptive nature of the data need to be considered as long as the absence of a control group to compare outcomes. The lack of translational correlative studies that could help to better understand MPM biology is another issue to consider. In addition, despite the analysis of the subsequent therapies after PD is outside the purpose of our study, their difference should be considered as they could affect the validity of OS reporting. In our study, inter-patients’ variability is high since patients have not been selected prospectively; however, MPM being a rare cancer type, and considering that no biomarker-driven patients selection could be currently applied outside of clinical trials, we believe that data from clinical practice are very helpful for designing future studies with a higher level of scientific evidence and could be hypothesis-generating. 

## 5. Conclusions

In conclusion, a trimodality four-step therapeutic approach in patients with early stage (I and II) MPM warrants further investigation, since we found it to be feasible in clinical practice and we foresee promising clinical outcomes. Our center experience showed no highest perioperative mortality and prolonged OS and PFS in a retrospective case series of 17 patients. Larger and prospective trials, including control groups, are needed to confirm clinical benefit and feasibility of this four-step approach.

## Figures and Tables

**Table 1:** Patients’ baseline characteristics.

Age, Median (Range)	66 (58–73)
Age category, *n* (%)	
<70	15 (88.2)
≥70	2 (11.8)
Sex, *n* (%)	
Male	12 (70.6)
Female	5 (29.4)
ECOG PS, *n* (%)	
0	5 (29.4)
1	10 (58.8)
2	2 (11.8)
Smoking status, *n* (%)	
Current/former	15 (88.2)
Never	2 (11.8)
Asbestos exposure, *n* (%)	
Yes	12 (70.6)
No	5 (29.4)
Clinical stage, *n* (%)	
I	14 (82.4)
II	3 (17.6)
Histology, *n* (%)	
Epithelioid	15 (88.2)
Sarcomatoid	0 (0)
Biphasic	2 (11.8)

ECOG PS: performance status according to Eastern Cooperative Oncology Group.

## Data Availability

The datasets used and/or analyzed during the current study are available from the corresponding author on reasonable request.

## Supplementary Materials

The following supporting information can be downloaded at: https://www.mdpi.com/article/10.3390/cancers14010142/s1, Figure S1: Patients flow and data collection, Figure S2: PFS (**A**) and OS (**B**) Kaplan-Meier curves by stage (1A vs. 1B/2).
