# Supplementary material for: A Trimodality, Four-Step Treatment including Chemotherapy, Pleurectomy/Decortication and Radiotherapy in Early-Stage Malignant Pleural Mesothelioma: A Single-Institution Retrospective Case Series Study
Source: Cancers (Basel). 2021 Dec 29;14(1):142. doi: 10.3390/cancers14010142 (PMC8749971; doi:10.3390/cancers14010142)
Supplement: Supplementary file 1 [file cancers-14-00142-s001.zip › cancers-1472763-supplementary.pdf]

Supplementary materials: A Trimodality, Four-Step Treatment Including Chemotherapy, Pleurectomy/Decortication and Radiotherapy in Early-Stage Malignant Pleural Mesothelioma: A Single-Institution Retrospective Case Series Study

Giovanni Vicidomini, Carmina M. Della Corte, Antonio Noro, Raimondo Di Liello, Salvatore Cappabianca, Alfonso Fiorelli, Valerio Nardone, Gaetana Messina, Giuseppe Viscardi, Angelo Sangiovanni, Riccardo Monti, Marina Accardo, Floriana Morgillo, Fortunato Ciardiello, Renato Franco 4 and Mario Santini

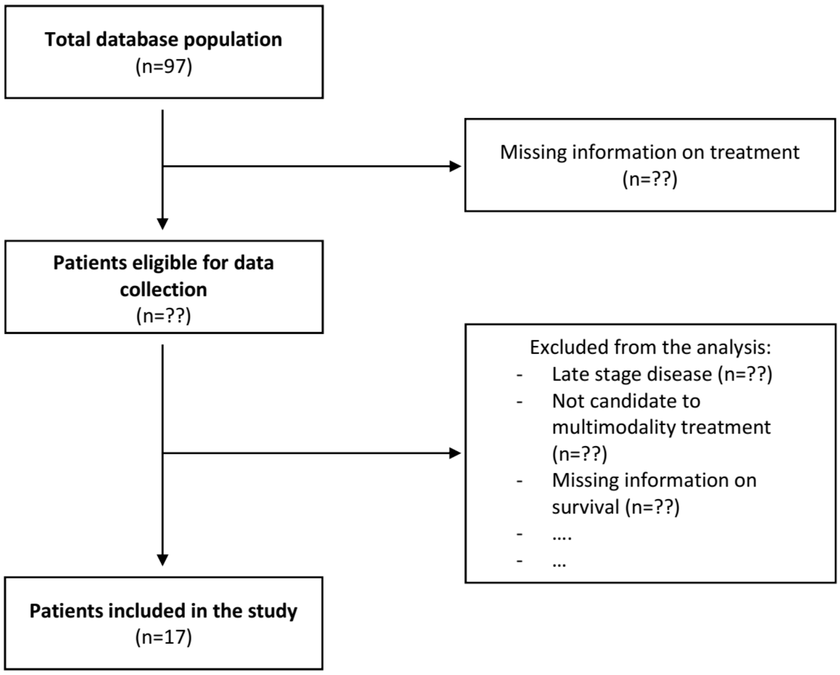

Figure S1. Patients flow and data collection.

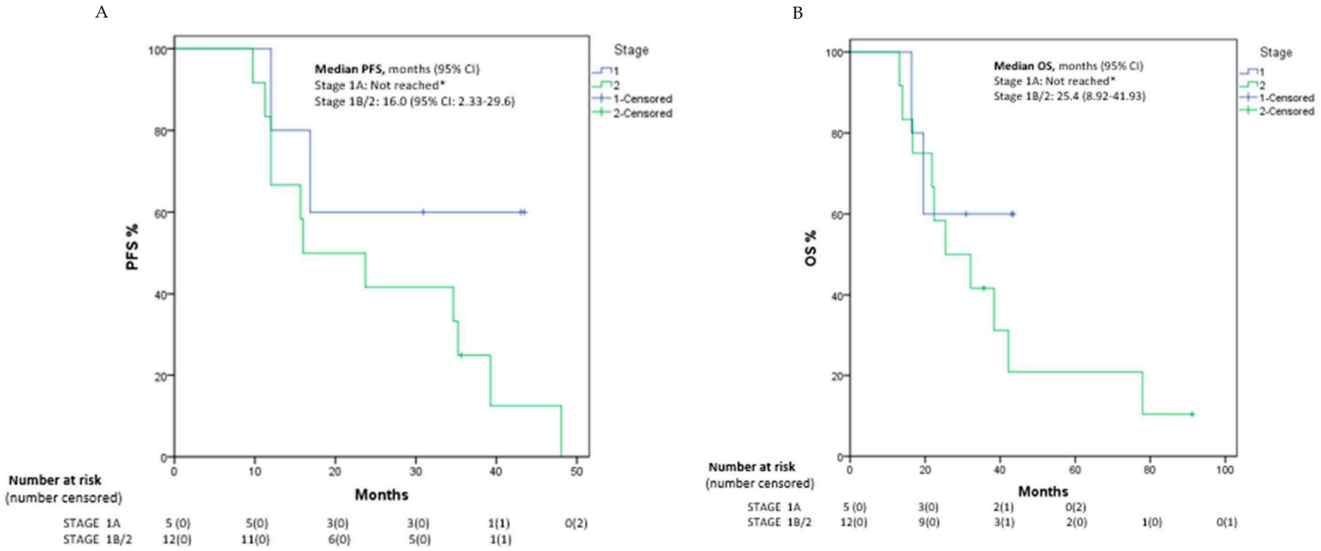

Figure S2. PFS (**A**) and OS (**B**) Kaplan-Meier curves by stage (1A vs. 1B/2).
